# Clinical experience with the new double aripiprazole depot regime in a psychiatric hospitalization unit

**DOI:** 10.1192/j.eurpsy.2022.1883

**Published:** 2022-09-01

**Authors:** Á. Moleón Ruiz, R. Tenorio Villegas, A. Gómez Rebollo

**Affiliations:** Hospital Juan Ramón Jiménez, Psychiatry, Huelva, Spain

**Keywords:** relapse, schizophrenia, aripiprazole, LAI

## Abstract

**Introduction:**

Nowadays, relapses are typical in patients with schizophrenia and may have serious implications due to most of them are caused by a lack of adherence to treatment.Therefore, numerous long-acting injectable antipsychotics (LAI) have been developed as aripiprazole depot who need a proper oral supplementation. Since November 2020, FDA approved a new treatment indication with a double injection start and a single dose of 20mg of oral aripiprazole, with the aim of avoiding oral supplementation during the next 14 days.

**Objectives:**

The purpose of our study is to expose our experience with the new double injection start with aripiprazole LAI, regarding the rehospitalization rate.

**Methods:**

A prospective study (n=17 patients) has been developed between November 2020 and October 2021 with the purpose of studying the rehospitalization and antipsychotic adherence after the new guideline of aripiprazole

**Results:**

After an 11-month-follow-up, it is noticed a 65% non-rehospitalization rate and a 76% proper treatment persistence rate. The 94% did not present any kind of side effects, only one patient had a case report of bullous pemphigoid. Regarding the concomitant use of other antipsychotics, 82% of the patients remained in monotherapia. The average stay time was shortened (11 days) regarding the standard dose (14 days).

**Conclusions:**

The new LAI regimen is well tolerated in our patients obtaining a high treatment persistence after several months of hospital discharge. It was already possible to shorten the time of rehospitalization, not having to add oral aripiprazole for 14 days. Most patients were discharged are on antipsychotic monotherapy and have not been rehospitalized in the short term.

**Disclosure:**

No significant relationships.

